# Uptake and perceptions of voluntary medical male circumcision among HIV-negative men in serodiscordant relationships in Zambia (2012–2015)

**DOI:** 10.1371/journal.pone.0309295

**Published:** 2024-11-08

**Authors:** Kalonde Malama, Susan Allen, Rachel Parker, Mubiana Inambao, Tyronza Sharkey, Amanda Tichacek, Kristin M. Wall, William Kilembe

**Affiliations:** 1 Center for Family Health Research in Zambia, Rwanda Zambia HIV Research Group, Lusaka, Zambia; 2 Ingram School of Nursing, Faculty of Medicine and Health Sciences, Mcgill University, Montreal, Quebec, Canada; 3 Rwanda Zambia HIV Research Group, School of Medicine, Emory University, Atlanta, Georgia, United States of America; 4 Center for Family Health Research in Zambia, Rwanda Zambia HIV Research Group, Ndola, Zambia; 5 Department of Epidemiology, Rollins School of Public Health, Emory University, Atlanta, Georgia, United States of America; University of Ottawa, CANADA

## Abstract

Voluntary medical male circumcision (VMMC) is a recommended HIV prevention strategy that few studies have promoted to HIV-negative men in serodiscordant relationships. We conducted a cross-sectional study on uptake and perceptions of VMMC among serodiscordant couples between 2012 and 2015. Heterosexual couples attending couples voluntary counselling and testing for HIV who had discordant results (M-, F+) were referred for VMMC. At least one month after counselling and referral, 343 men were surveyed on uptake and perceptions of VMMC. A subset of 134 uncircumcised men responded to another survey assessing their intention to uptake VMMC and reasons for not getting circumcised. Forty percent (n = 62) of men eligible for VMMC either up took (n = 22) or planned to uptake circumcision (n = 40). The most cited reasons for not getting circumcised were the inability to get time off work (34%) and culture/traditions (26%). These findings support integrated approaches, pairing evidence-based HIV prevention interventions such as couples voluntary counselling and testing with VMMC, and targeting men at highest risk for HIV. Additional counselling may be needed for couples whose cultural backgrounds do not support VMMC.

## Introduction

In 2007, the World Health Organisation (WHO) and UNAIDS recommended the scale up of voluntary medical male circumcision (VMMC) to cover 80% of HIV-negative uncircumcised men aged 15–49 in 15 HIV-endemic countries in East and Southern Africa by 2025 [1]. The recommendation was based on data from three randomised controlled trials [2] including evidence from East Africa showing that VMMC reduced the risk of HIV acquisition by 60% in circumcised men compared to uncircumcised men [3, 4]. The 15 priority countries were selected based on their high prevalence of HIV and low prevalence of VMMC [5]. In 2021, fourteen years after the initial recommendation, UNAIDS reports that the 15 priority countries for VMMC are falling behind on circumcision targets [6].

Among the UNAIDS priority countries is Zambia, where—during the scaleup—the number of voluntary medical male circumcisions performed rose from 2,758 in 2008 to 482,183 in 2018 [7]. Despite the VMMC scaleup initiative, barriers to circumcision uptake exist. One such barrier is that VMMC in Zambia usually occurs during a short time window that targets school boys and restricts service access to the adult population; specifically, 75% of circumcisions taking place during VMMC campaigns in only three months out of the year—coinciding with school holidays [8]. Integrating VMMC into routine healthcare services is mentioned as a solution in Zambia [8]. Studies over the years have also reported a reluctance by HIV-negative men to get circumcised and long waiting times for men seeking services [9]. According to Weiss, Zulu [10], this reluctance to get circumcised can be circumvented by providing comprehensive HIV risk reduction information emphasising VMMC but also highlighting other HIV prevention interventions. Hewett, Nalubamba [11] corroborate the need to integrate VMMC with HIV counselling, testing and risk reduction information to improve uptake of VMMC.

Heterosexual transmission accounts for the majority of HIV infections in SSA [12]. It follows that HIV serodiscordant couples—in particular, couples where the man is HIV-negative and the woman is living with HIV—would be prime candidates for VMMC due to the risk of exposure faced by the man if their partner is not virally suppressed. Couples Voluntary HIV Counselling and Testing (CVCT) is a WHO-recommended HIV prevention intervention that reduces HIV by 63% among discordant couples not on antiretroviral treatment (ART) and 79% among discordant couples who are on ART [13]. CVCT reduces HIV by providing accurate knowledge of partner status [14, 15], reducing the incidence of sexually transmitted infections [16, 17], and increasing awareness that serodiscordance is possible [18]. Integrating VMMC and CVCT for HIV-negative men in discordant couples would represent a change in approach from mass circumcising HIV-negative men to targeting men at greatest risk with two effective HIV prevention interventions.

Despite the potential benefits of combining VMMC and CVCT, there is a dearth of studies looking at the linkage of HIV discordant couples to VMMC services. On this basis, we conducted a cross-sectional study assessing the uptake and perceptions of VMMC among HIV-negative Zambian men and their partners living with HIV. Our findings could inform current VMMC strategies in Zambia and the 14 other UNAIDS priority countries.

## Methods

### Study design

This was a sub-study nested in a larger programme providing CVCT in government clinics [13, 19–21]. Among the couples attending baseline (Month 0) CVCT, men and women testing HIV-positive were referred for ART assessment in accordance with Zambian Ministry of Health guidelines; couples testing concordant HIV-negative (M-F) and discordant (M-, F+ or M+, F-) were asked to return for follow-up at Month 1, Month 3 and quarterly thereafter. At Month 0, couples received CVCT with an emphasis on HIV risk reduction messaging, including—for discordant (M-, F+ couples)—information on the benefits of VMMC and a referral for circumcision at the nearest facility providing the service.

For this sub-study, the Center for Family Health Research in Zambia (formerly the Zambia Emory HIV Research Project) trained healthcare workers in 55 government clinics [22] to conduct a cross sectional survey from June 2012 to June 2015. The survey assessed the uptake and intentions to uptake VMMC at a follow-up visit at least one month after referral, and the perceptions of VMMC among HIV-negative men and their female partners in serodiscordant relationships. Couples were selected to participate in the sub-study using convenience sampling based on their presence at the clinic, willingness to enrol, and meeting the inclusion criteria.

**Fig 1:**
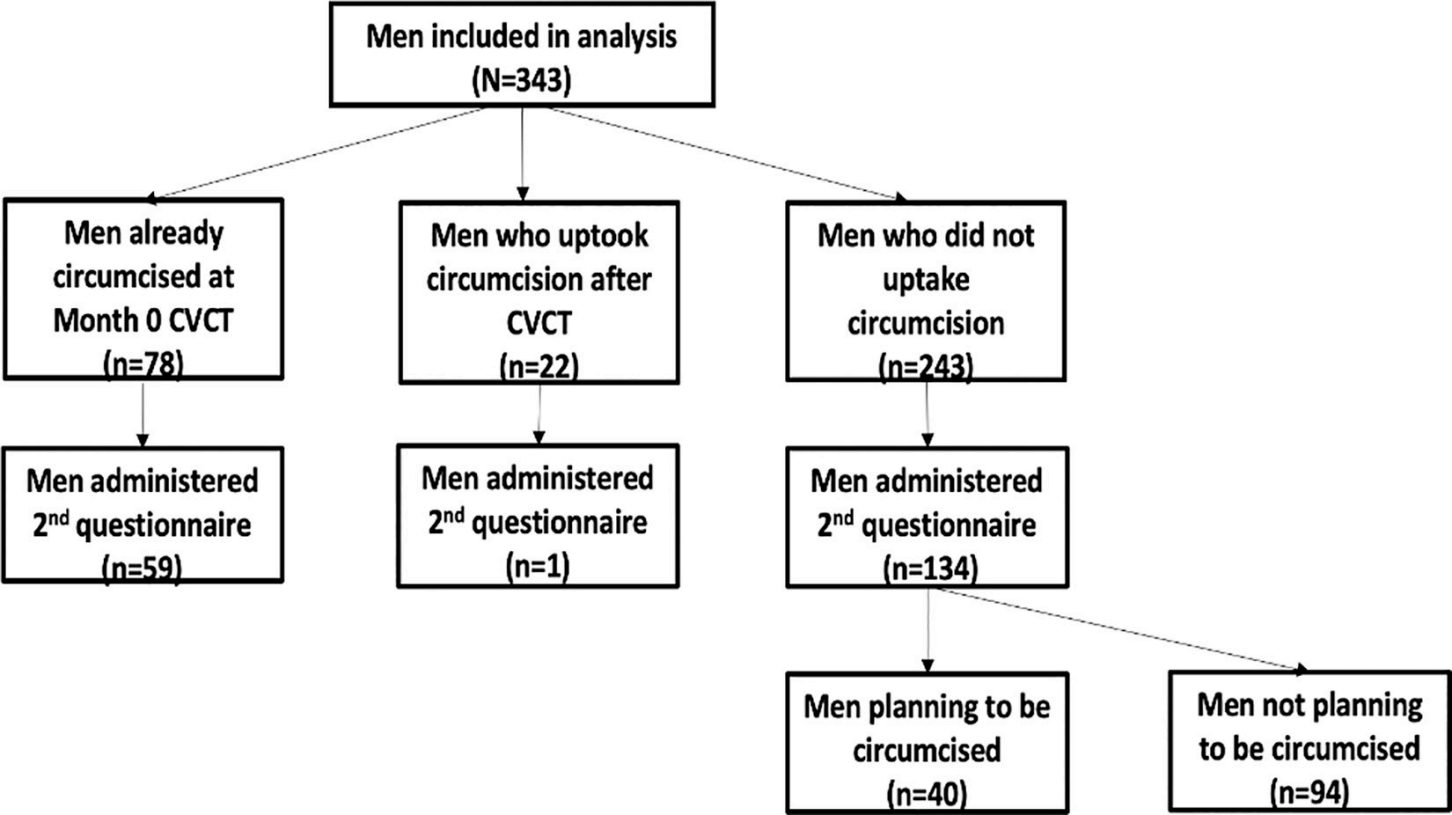
Flowchart of study participants (N = 343).

Overall, 433 men were administered the questionnaire, which contained questions developed after pre-administered focus group discussions with serodiscordant couples enrolled in the larger CVCT cohort. Ninety questionnaires were excluded due to improper consent (n = 46), incorrect serostatus (n = 18), incomplete/missing information (n = 12), mismatched identity (n = 1) and indeterminate circumcision status (n = 13). A total of 343 men met the eligibility criteria of being aged 18 and over, providing written consent to participate in the study, testing negative for HIV and having a partner living with HIV (Fig 1). The 343 men were administered an initial questionnaire that collected sociodemographic information and assessed uptake of circumcision. A subset of men (n = 194) and their female partners (n = 155) were selected via convenience sampling to complete a second questionnaire, which asked participants who did not get circumcised whether they were planning to get circumcised; assessed the perceived benefits of VMMC among circumcised and uncircumcised couples; and collected reasons for not getting circumcised among men who did not uptake VMMC.

### Study setting

This cross-sectional study was conducted in 55 Zambian government clinics spread across Lusaka, Southern and Copperbelt provinces. The clinics were primary and secondary healthcare facilities based in urban and peri-urban areas of the three provinces. Lusaka, the capital city, has a population of over three million and represents the most urbanised of the three provinces [23]. The Copperbelt province, a mining hub of the country, has a population of 2.5 million and has levels of urbanisation similar to Lusaka [23]. Southern province, mainly known for farming and tourism, is the least urbanised of the three provinces with a population of just under 2 million people [23]. All three provinces have high HIV prevalence; Lusaka has the highest with 15.7%, followed by the Copperbelt (13.8%) and Southern (13.3%) provinces [24].

### Ethics

This study was approved by the University of Zambia Biomedical Research Ethics Committee and the Emory University Institutional Review Board (IRB00001131). All study documents, including the protocol, consent forms and questionnaires were reviewed and approved by the above bodies. Couples who agreed to participate provided written informed consent to enrol in the study in a language they were comfortable with, i.e., English, Bemba or Nyanja.

### Study procedures

When couples arrived at their respective health facility for their follow-up visit, they were seen by nurses or nurse/counsellors trained in psychosocial couples’ HIV counselling and testing, and discordant couples (M-, F+) were invited to participate in the VMMC sub-study. Nurse/counsellors—trained to reduce interviewer bias by assuring participants that their responses would not attract stigma or discrimination—administered the study questionnaire assessing uptake and perceptions of VMMC to all participants on a one-to-one, confidential basis. Male and female partners were administered the survey separately. Questionnaires were given in English or a local language that participants were more comfortable with.

### Measures

#### VMMC uptake

The first outcome assessed in this study was the uptake of VMMC after CVCT. We determined uptake to have taken place if uncircumcised men who received a referral for VMMC at Month 0 responded “yes” to the following question on the survey: “Are you now circumcised” at Month 1?

#### Intention to uptake VMMC

We asked a subset of men who did not get circumcised after receiving a referral for VMMC whether they intended to get circumcised. Men responding “yes” were categorised as planning to get circumcised.

#### Perceptions of VMMC

Couples who returned for follow-up fell into three categories: 1) those who were circumcised before/after baseline; 2) those who were not circumcised but planning to; 3) and those who had no intention of getting circumcised. In all three configurations, men and their female partners were asked whether they were aware of any benefits of VMMC. Participants who responded “yes” were then asked to mention what benefits they were aware of. As participants mentioned benefits, nurse/counsellors recorded responses.

#### Reasons for not getting circumcised

Uncircumcised men returning for follow-up indicated that they were planning to get circumcised or had no intention of doing so. Men in both categories were asked to provide reasons why they did not uptake VMMC after the referral they received at Month 0. Nurse/counsellors noted down these reasons as participants mentioned them.

#### Sociodemographics

Nurse/counsellors collected sociodemographic data including age, education, occupation, marital status and ethnic group.

### Statistical analysis

We performed a descriptive analysis of the data collected during the VMMC sub-study. Means and standard deviations were calculated to summarise continuous variables. Frequencies and percentages were employed to summarise categorical variables. We performed chi-squared tests (or Fisher’s exact tests) to determine if there were any statistically significant differences in sociodemographics, perceptions of VMMC and reasons for not getting circumcised between circumcised and uncircumcised men. Chi-squared (or Fisher’s exact) tests were also employed to determine if perceptions of VMMC and reasons for not getting circumcised differed significantly between women whose spouses were circumcised, planning to get circumcised or not planning to be circumcised. All statistical tests were conducted using SAS 9.4 (Cary, NC).

## Results

Of the 343 men enrolled in this study, 23% (n = 78) were already circumcised before attending CVCT at baseline. Six percent (n = 22) of men took up VMMC after being jointly counselled and tested for HIV with their female partners. Of the remaining 243 uncircumcised men, a subset (n = 134) responded to a second questionnaire in which 30% (n = 40) indicated that they planned to get circumcised in the future; while 70% (n = 94) reported no intention of getting circumcised in the future (Fig 1). Among the 100 men circumcised before or after CVCT, 60 were administered the second questionnaire, of whom 80% (n = 48) were accompanied by female partners who also responded to the questionnaire (Fig 1). Of the 40 men planning to get circumcised, 88% (n = 35) completed the second questionnaire with their female partners (Fig 1). Seventy-seven percent of men not planning to be circumcised (n = 72) attended follow-up visits with their female partners, who also completed the questionnaire (Fig 1).

Table 1 shows sociodemographic characteristics of all 343 HIV-negative men enrolled in this study, stratified by circumcision status—that is, circumcised before CVCT, circumcised after CVCT, and uncircumcised. The mean age of participants was 38 (standard deviation: 11). Over half of participants (52%) came from the Copperbelt province of Zambia, followed by Lusaka (41%) and Southern Province (7%). Most men (91%) were married and attained either secondary school (65%) or primary school (24%) education. The two most represented ethnic groups were Bembas (28%) and Lozis (10%), and the two most represented occupations were skilled (35%) and unskilled (24%) manual labour. Differences in circumcision prevalence or uptake were noted by age (43% of circumcised men vs. 29% of uncircumcised men were aged < = 31 years, *P*<0.05); region (61% of circumcised men vs. 48% of uncircumcised men resided in the Copperbelt, *P*<0.05) and ethnic group (25% of circumcised men were Lozi vs. 3% of uncircumcised men, *P*<0.001).

**Table 1:** Characteristics of HIV-negative men in serodiscordant relationships by circumcision status (N = 343).

	Circumcised at baseline (N = 78)		Circumcised after baseline (N = 22)		Did not uptake circumcision (N = 243)		*P*-value*
	n	%	n	%	n	%	
**Age**							0.042
< = 31	35	45	8	36	71	29	
32–40	23	29	7	32	85	35	
> = 41	20	26	7	32	87	36	
**Region**							0.031
Copperbelt	47	60	14	64	117	48	
Lusaka	25	32	8	36	109	45	
Southern Province	6	8	0	0	17	7	
**Marital status** ^**1**^							0.837
Pre-marital (courting)	5	6	0	0	15	6	
Married monogamous	66	85	21	95	205	85	
Married polygamous	5	6	1	5	14	6	
Cohabitating (but unmarried)	2	3	0	0	7	3	
**Ethnic group** ^**2**^							<0.001
Bemba	16	31	6	29	50	29	
Nyanja/Chewa	3	6	0	0	13	8	
Lozi	21	41	4	19	8	5	
Tonga	0	0	1	5	19	11	
Other	11	22	10	48	82	48	
**Education**							0.506
None	1	1	0	0	4	2	
Primary	20	26	6	27	58	24	
Secondary	48	62	12	55	162	67	
Tertiary	9	12	4	18	19	8	
**Occupation** ^**3**^							0.291
Professional	9	12	2	10	16	7	
Agriculture	5	6	3	15	15	6	
Skilled manual labour	25	32	11	55	83	34	
Unskilled manual labour	21	27	1	5	59	24	
Sales/ Service	5	6	2	10	36	15	
Other	13	17	1	5	32	13	

1. 2 missing marital status

2. 99 missing ethnic group

3. 4 missing occupation

*P-values compare men circumcised at baseline versus men who did not uptake circumcision

Table 2 shows the benefits of VMMC cited by a subset of men who were either circumcised (n = 60), planning to get circumcised (n = 40) or not planning to get circumcised (n = 94). Virtually all men (98%) who were circumcised before or after CVCT reported knowing of at least one benefit of VMMC compared to 82% of men who were uncircumcised (*P*<0.001). The most commonly cited benefits were related to sexual health and hygiene; men across all three circumcision categories perceived VMMC to be beneficial in reducing the risk of STIs (80%) and HIV (71%), and the enhancement of hygiene (59%). There were significant differences between circumcised and uncircumcised men when citing benefits of VMMC making putting on a condom easier and increasing social acceptability. A greater proportion of circumcised men cited the benefit of increased social acceptability as a result of VMMC compared to uncircumcised men (25% vs 13%, p<0.05). A smaller proportion of circumcised men cited the benefit of VMMC making condom use easier compared to uncircumcised men (45% vs 28%, p<0.05).

**Table 2:** Health benefits of circumcision identified by HIV-negative men in serodiscordant relationships, stratified by circumcision status.

	Circumcised (n = 60)		Planning (n = 40)		Not Planning (n = 94)		P-value
	n	%	n	%	n	%	
Know of any benefits of VMMC? ^1^	58	98	39	98	71	76	0.001
Reduces risk of HIV acquisition	51	88	34	87	57	80	0.065
Reduces risk of STI acquisition	48	83	34	87	59	83	0.756
Hygienic	43	74	32	82	47	66	0.092
Reduces risk of cervical cancer in partners	18	31	21	54	23	32	0.274
Reduces the risk of penile cancer	16	28	15	38	19	27	0.153
Makes putting a condom on easier	14	24	9	23	7	10	0.031
Increases my sexual pleasure	13	22	4	10	4	6	0.247
Increases the sexual pleasure of my partner	9	16	5	13	1	1	0.143
Increased sexual desirability	6	10	5	13	3	4	0.701
Increases social acceptability	7	12	2	5	1	1	0.043

1: One missing response

*P-values compare circumcised and uncircumcised men

Perceptions that VMMC made putting on a condom easier and increased sexual pleasure for males were the most cited among men who were planning to be circumcised compared to men not planning to be circumcised (23% vs 8% and 17% vs 5%, respectively, *P*<0.01). Additional differences were noted between the two groups in the proportion reporting increased sexual pleasure of female partners (14% vs 1%, *P*<0.001); increased sexual desirability (11% vs 4%, *P*<0.05) and increased social acceptability (9% vs 1%, *P*<0.05).

Table 3 summarises the reasons why men did not get circumcised, stratified by men planning to get circumcised and men not planning to get circumcised. The primary reason for not getting circumcised by men who planned to get circumcised was their inability to get time off work (64%). Among men with no intention to get circumcised the major deterrent cited was cultural or traditional values (42%), with 30% also citing not being able to get time off work. Concerns about pain following circumcision (36% of circumcised men; 29% of uncircumcised men), the abstinence period following the procedure (28% of circumcised men; 14% of uncircumcised men), the safety of the procedure (15% of circumcised men; 18% of uncircumcised men), and impact on sexual pleasure (15% of circumcised men; 12% of uncircumcised men) were cited by both groups. Few men (15% of circumcised men; 5% of uncircumcised men) reported concerns that VMMC might cause impotence though men planning to be circumcised were more likely to mention this. Men planning to be circumcised were also more likely to mention being unable to cover the cost of the service and transportation. Few men in either group reported being discouraged by their wives or religious reasons.

**Table 3:** Reasons for net getting circumcised reported by HIV-negative men planning and not planning to get circumcised (N = 134).

	Planning to get circumcised (N = 40)		Not planning to get circumcised (N = 94)	
	n	Col%	n	Col%
Unable to get time off from work	25	64	20	30
Unable to afford the cost of the service	5	13	2	3
Unable to afford transportation to health care center offering VMMC	4	10	0	0
Concerns about pain during/ following the procedure	14	36	19	29
Abstinence period after the procedure	11	28	9	14
Concerns with safety of the procedure	6	15	12	18
Concerns about the impact of MMC on sexual pleasure	6	15	8	12
Concerns that MMC might cause impotence	6	15	3	5
Cultural/traditional reasons	7	18	28	42
Discouraged by wife/partner	3	8	4	6
Religious reasons	0	0	6	9

## Discussion

This study analysed uptake and perceptions of VMMC among HIV-negative men in serodiscrodant relationships and found that 8% of men were circumcised after referral from CVCT, and a further 30% of surveyed VMMC-eligible men intended to get circumcised. Our findings demonstrate that it is possible to leverage CVCT, an evidence-based HIV prevention intervention, to identify high-risk couples for HIV and link them to VMMC. Previous research in Zambia showing that female partner acceptance of VMMC translates into higher acceptance among men supports our suggestion for CVCT as a precursor to VMMC referral based on our finding that 40% of surveyed men in discordant relationships who were eligible for VMMC either up took or planned to uptake VMMC after receiving CVCT [25]. Counsellor-facilitated discussion of VMMC, provided to discordant couples, could foster the partner support required to overcome some of the logistical and recovery-related concerns around VMMC cited by men in our study. Alongside CVCT, VMMC programmes could incorporate other evidence-based strategies that have been effective at improving uptake in UNAIDS priority countries, including mobile clinics, home-based services and facility-based VMMC services with comprehensive sexual health messaging [26].

Men who did not get circumcised but indicated a desire to do so in the future, cited inability to get time off work and concerns about procedural pain as major barriers. A potential solution to the cited inability to get time off work could be promoting VMMC in the workplace, which is a recommended strategy for high-risk populations [27]. Off- hour clinics that prioritise men at highest risk of HIV could help resolve the logistical barrier of men not being able to leave work to access VMMC; off-hours could also serve the benefit of decongesting VMMC services, which are hampered by clinic overcrowding [9]. Reports of pain as a barrier are consistent with findings from Schenk, Friedland [28] that men who underwent circumcision in Zambia and Eswatini were surprised by how much pain they felt during administration of anaesthesia and after surgery. These findings indicate a need for better health education prior to VMMC to set realistic expectations around procedural pain.

Among men not planning to be circumcised, 42% cited cultural/traditional reasons. What is clear from our study and the literature is that VMMC programmes in Zambia compete with cultural norms that—with the exception of a few tribes and religions—do not promote circumcision. According to Jones, Rodriguez [29], close to 80% of HIV-negative Zambian men disclosed no interest in getting circumcised in 2013. Efforts to reach men, such as those in our study, in serodiscordant unions and no interest in getting circumcised after CVCT, must consider doing so while bearing in mind the existing norms among Zambian men that shape their decision making. Targeted community engagement could be an effective strategy to overcome opposition to circumcision based on tradition or religion; for instance, traditional leaders and faith-based leaders could be engaged to deliver VMMC health messaging. A recent study from Zambia suggests that leveraging different community structures and channels of communication—including social media, radio, television, community health workers, neighbourhood health committees, schools, churches and other community spaces—could be a way of increasing VMMC uptake [30]. Additionally, a Malawian study among traditionally circumcising and uncircumcising tribes cautions that any communication must incorporate feedback from the receiving audience to the health promoters [31].

Unfortunately, our study did not ask men what exact traditions/customs prevented them from getting circumcised. However, sociocultural stigma against circumcised men is evident in the literature, where Chiringa, Ramathuba [32] state that circumcised men in Zimbabwe were labelled *promiscuous*, *shameful* and *worthless*. Conversely, Rennie, Gilbertson [33] highlight experiences of uncircumcised males being stigmatised during the epoque of VMMC scaleup, where being circumcised is viewed as being clean and caring about others. In a sense, the risk of HIV infection in endemic African countries brings with it hitherto uncommon practices such as VMMC, which upset existing cultural norms. African men are experiencing a shift from viewing circumcision as a traditional/religious practice for certain people towards circumcision seen as a public health intervention for HIV-negative men [34]. Such a transition requires time and a careful consideration of cultural context by VMMC programmes in Africa.

Further, counselling models could invite previously circumcised men to speak at healthcare facilities to their uncircumcised counterparts about the experience of circumcision. This could alleviate concerns around the pain of the procedure and the extensive periods of time off work that our study participants expressed. A similar model was employed in Zambia by using previous clients at family planning clinics to promote long-acting contraceptives to eligible women, which led to a higher uptake of contraception [19]. Although most men planning to get circumcised in our study perceived VMMC to reduce HIV risk, around a third cited abstinence after the procedure as a concern. Counselling models should therefore educate men around the risks of resuming sex before healing as this practice could heighten their HIV risk [35]. Counselling messages should also address potential risk compensation, resulting from men and their partners engaging in riskier sexual behaviour in the belief that VMMC eliminates HIV risk [36, 37].

Our study findings must be considered among certain limitations. Firstly, our findings are subject to social desirability bias because uncircumcised participants were likely to respond positively to questions regarding their intention to get circumcised in the future in order to please the interviewers. However, this bias was minimised by training the nurse/counsellors administering the questionnaires to reassure participants that answering truthfully would not lead to any stigma or judgement. Secondly, we measured uptake one month after participants received counselling; this timeframe may have been too short as participants might have required more time to reflect on counselling messages before deciding to get circumcised. Thirdly, our findings are subject to sampling bias due to our use of convenience sampling to recruit study participants. Fourth, we were limited by a small sample size of participants who got circumcised and therefore could not conduct a multivariable regression analysis measuring the factors associated with uptake of circumcision during the study. Lastly, when this study took place, alternative circumcision methods such as the PrePex device—which is worn on the penis and necrotises the foreskin—were not widely available in Zambia. The option of PrePex—which was 99% effective at circumcising Rwandan men, and was highly acceptable among Botswanan men, who reported manageable pain—could have alleviated the fear of pain cited as a major barrier to circumcision by men in our study [34, 35].

Despite its limitations, our study makes a valuable contribution to the limited literature on VMMC uptake among the high-risk population of HIV-negative men in discordant relationships. Moreover, lessons learnt from promoting VMMC in HIV counselling and testing clinics are valuable to inform the integration of VMMC into routine healthcare service delivery since the majority of VMMC uptake in WHO/UNAIDS countries is still attributed to seasonal circumcision campaigns [26]. A recent study analysing Zambian demographic health survey data from 2008 (around the time of VMMC scaleup) to 2017 revealed, somewhat surprisingly, that VMMC did not reduce HIV among males aged 15–49 [38]. This finding—rather than being an indictment of VMMC as an HIV prevention strategy—could be viewed as a call to move away from circumcising HIV-negative men *en masse* and towards circumcising those at highest risk of acquiring HIV.

## Conclusion

Our study demonstrated the potential benefit of integrating CVCT with messaging and referral for VMMC by showing that 40% of uncircumcised HIV-negative men in serodiscordant relationships either up took or planned to uptake circumcision after receiving CVCT with a referral for VMMC. Moreover, participants in our study were highly aware of the potential benefits of VMMC. Further studies employing more rigorous study designs such as longitudinal surveys will be required to demonstrate the effectiveness of an integrated CVCT/VMMC model in reducing HIV risk. With many HIV prevention approaches now geared towards targeting populations at highest risk, our findings could inform VMMC strategies in similar settings. In particular, our findings from Zambia are relevant to the other 14 UNAIDS/WHO priority countries that are undertaking huge circumcision campaigns. Once WHO/UNAIDS scaleup targets are eventually met and funding cycles come to a close, healthcare systems will need to employ more sustainable and cost-effective approaches to VMMC. In particular, integrating VMMC into established HIV counselling and testing services, with counselling messages adapted to address barriers to VMMC such as fear of pain and inability to get time off work. We recommend that men at highest risk of acquiring HIV be prioritised when VMMC services are limited.

## Supporting information

S1 Dataset (XLSX)
